# Unchanged content of oxidative enzymes in fast-twitch muscle fibers and 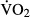 kinetics after intensified training in trained cyclists

**DOI:** 10.14814/phy2.12428

**Published:** 2015-07-07

**Authors:** Peter M Christensen, Thomas P Gunnarsson, Martin Thomassen, Daryl P Wilkerson, Jens Jung Nielsen, Jens Bangsbo

**Affiliations:** 1 Department of Nutrition, Exercise and Sports, Section of Integrated Physiology, University of CopenhagenCopenhagen, Denmark; 2 Team Danmark (Danish Elite Sport Organization)Copenhagen, Denmark; 3 Sport and Health Sciences, St Luke’s Campus, University of ExeterExeter, UK

**Keywords:** V˙O_2_ kinetics, high intensity training, interval training, type I fibers, type II fibers

## Abstract

The present study examined if high intensity training (HIT) could increase the expression of oxidative enzymes in fast-twitch muscle fibers causing a faster oxygen uptake (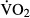) response during intense (INT), but not moderate (MOD), exercise and reduce the 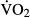 slow component and muscle metabolic perturbation during INT. Pulmonary 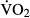 kinetics was determined in eight trained male cyclists (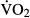-max: 59 ± 4 (means ± SD) mL min^−1^ kg^−1^) during MOD (205 ± 12 W ∼65% 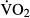-max) and INT (286 ± 17 W ∼85% 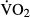-max) exercise before and after a 7-week HIT period (30-sec sprints and 4-min intervals) with a 50% reduction in volume. Both before and after HIT the content in fast-twitch fibers of CS (*P* < 0.05) and COX-4 (*P* < 0.01) was lower, whereas PFK was higher (*P* < 0.001) than in slow-twitch fibers. Content of CS, COX-4, and PFK in homogenate and fast-twitch fibers was unchanged with HIT. Maximal activity (*μ*mol g DW^−1^ min^−1^) of CS (56 ± 8 post-HIT vs. 59 ± 10 pre-HIT), HAD (27 ± 6 vs. 29 ± 3) and PFK (340 ± 69 vs. 318 ± 105) and the capillary to fiber ratio (2.30 ± 0.16 vs. 2.38 ± 0.20) was unaltered following HIT. 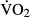 kinetics was unchanged with HIT and the speed of the primary response did not differ between MOD and INT. Muscle creatine phosphate was lower (42 ± 15 vs. 66 ± 17 mmol kg DW^−1^) and muscle lactate was higher (40 ± 18 vs. 14 ± 5 mmol kg DW^−1^) at 6 min of INT (*P* < 0.05) after compared to before HIT. A period of intensified training with a volume reduction did not increase the content of oxidative enzymes in fast-twitch fibers, and did not change 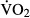 kinetics.

## Introduction

It has been known for decades that endurance training results in a faster increase in the pulmonary oxygen uptake (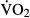) response in the initial phase of exercise (Hickson et al. 1978). During constant load exercise at intensities above the gas exchange threshold (GET) 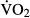 continues to rise at a slow rate, with training reported to reduce the magnitude of this ‘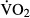 slow component’ (Jones et al. 2011). These alterations may be due to elevated muscle oxidative enzyme capacity and greater oxygen delivery (Jones and Poole 2005) as have been found in training studies using untrained subjects with a maximal 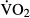 (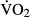-max) of ∼50 mL min^−1^ kg^−1^ (Saltin et al. 1976; Phillips et al. 1995; Shoemaker et al. 1996; Krustrup et al. 2004a). In the study of Krustrup et al. subjects performed high intensity training (1 min intervals) and it was reported that post-training leg 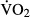 kinetics was faster during intense but not moderate exercise when compared to pretraining, respectively (Krustrup et al. 2004a). This may have been caused by adaptations in fast-twitch (FT) muscle fibers recruited during training, since these fibers have been reported to have lower oxidative maximal enzyme activity than slow-twitch (ST) muscle fibers (Essen et al. 1975; Essen-Gustavsson and Henriksson 1984; Schantz and Henriksson 1987). In support for training causing fiber type-specific adaptations, maximal activity of oxidative enzymes in FT muscle fibers can reach the same level as ST fibers in highly trained athletes (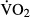-max: ∼70 mL min^−1^ kg^−1^) (Jansson and Kaijser 1977; Chi et al. 1983) and a training study with untrained subjects observed an increase in maximal activity in pools of FT fibers following intense training (Henriksson and Reitman 1976).

Training studies using untrained subjects typically result in several adaptations. Therefore, it cannot be determined if one adaptation (e.g., increase in oxidative enzyme capacity) is of importance for the faster rise in 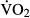 at the onset of exercise and the alterations in the 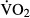 slow component when other adaptations also occur in response to training (e.g., increase in bulk oxygen delivery or reduced mean transit time at the capillary level due to increased capillary density). Instead, studying the effect of an altered training regime of trained subjects could potentially provide insight into what factors are important to improve the 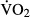 kinetics since fewer muscular and vascular adaptations are expected to occur. Furthermore, just a few studies have been conducted with trained athletes and some (Dufour et al. 2006; Christensen et al. 2011), but not all studies (Norris and Petersen 1998; Demarle et al. 2001), reported that 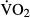 kinetics remained unaltered. However, comparison is difficult due to various designs used ranging from high-volume training for 8 weeks at moderate intensity at the start of the season in cyclists (Norris and Petersen 1998), 6 weeks in runners encompassing both moderate and intense bouts (∼90% 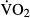-max in hypoxia or normoxia) (Dufour et al. 2006), to intense training (<5 min) either with a reduction in volume in soccer players for 2 weeks at the end of the season (Christensen et al. 2011) or a maintained training volume in runners for 8 weeks (Demarle et al. 2001).

Recruitment of FT fibers has been implicated in the development of the 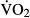 slow component (Barstow et al. 1996; Jones et al. 2011). Thus, it may be that an increased content of oxidative enzymes in FT fibers could reduce the 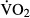 slow component in trained athletes since a high proportion of FT fibers has been associated with a large slow component (Barstow et al. 1996) and since FT fibers appear to have a lower maximal oxidative enzyme activity than ST fibers (Essen et al. 1975; Essen-Gustavsson and Henriksson 1984; Schantz and Henriksson 1987) unless vigorous training has been performed (Jansson and Kaijser 1977; Chi et al. 1983). Moreover, since oxidative enzymes are thought to be implicated in the rate of the rise in 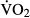 at the onset of exercise (Jones and Poole 2005), it may be that intense training targeting both ST and FT fibers is essential for eliciting faster 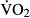 kinetics in trained athletes. This argument is supported by an augmented signaling for cascades involved in the synthesis of oxidative enzymes in well-trained cyclists (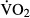-max: 68 mL min^−1^ kg^−1^) following repeated 30-sec sprinting compared with less intense exercise (Psilander et al. 2010). Also, in less trained subjects repeated 30-sec sprinting activated signal cascades involved in the synthesis of oxidative enzymes to a similar extent as long duration low intensity exercise (Little et al. 2010, 2011). The response of these signaling cascades increases in an intensity-dependent manner (Egan et al. 2010; Nordsborg et al. 2010), which could be related to recruitment of more muscle fibers. In runners subjected to intense training in combination with a marked lowering of low- and moderate intensity training (∼25–50%) maximal oxidative enzyme activity in muscle homogenate was maintained (Bangsbo et al. 2009; Iaia et al. 2009) which could be due to adaptations in FT fibers since detraining is known to lower activity of oxidative enzymes (Chi et al. 1983; Christensen et al. 2011). Thus, repeated 30-sec sprinting separated by ∼4 min of rest may be an effective training regime to cause increases in oxidative enzymes in FT fibers. The majority of studies in untrained subjects (Jacobs et al. 1987; MacDougall et al. 1998; Burgomaster et al. 2005, 2008; Gibala et al. 2006) did not evaluate fiber type-specific adaptations in oxidative enzymes, but recent studies do suggest that repeated 20–30 sec sprints can increase markers of oxidative capacity in both fiber types in untrained subjects (Shepherd et al. 2013; Scribbans et al. 2014). However, such evaluation has not been performed on trained individuals following intense training with 30-sec sprints (Bangsbo et al. 2009; Iaia et al. 2009; Christensen et al. 2011; Gunnarsson et al. 2012). Oxidative capacity is higher in ST than FT fibers (Essen et al. 1975; Essen-Gustavsson and Henriksson 1984; Schantz and Henriksson 1987, Shepherd), which means that the potential for improvements likely is greater in the FT fibers supported by similar capacity in the two fiber types in highly trained subjects (Jansson and Kaijser 1977; Chi et al. 1983).

A faster 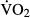 response is associated with reductions in the anaerobic contribution to the total energy turnover at the onset of exercise. This has been determined from muscle metabolites and blood lactate either as a result of performing prior exercise (Bangsbo et al. 2001; Krustrup et al. 2001) or performing exercise training either with low intensity and high volume (Phillips et al. 1995; Green et al. 2009) or high intensity and low volume (Burgomaster et al. 2006). However, limited knowledge exists about muscle anaerobic energy turnover after a period of high intensity training of trained subjects. Venous lactate when running at intensities below 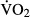-max was unchanged after 4 weeks of repeated 30-sec sprint training in moderately trained runners (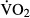-max: 55 mL min^−1^ kg^−1^) (Iaia et al. 2009) and after 6–9 weeks of combined aerobic high intensity training and repeated 30-sec sprinting in trained runners (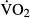-max: 61 mL min^−1^ kg^−1^) (Bangsbo et al. 2009), with both studies encompassing a reduction in weekly training distance. Changes in blood lactate likely reflect changes at the muscular level (Green et al. 2009) but neither of the studies evaluated changes in muscle lactate and creatine phosphate during exercise. Following 3 weeks with high-intensity training (8 × 5 min) added to the normal training, cyclists (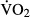-max: 65 mL min^−1^ kg^−1^) had lower muscle lactate but unchanged creatine phosphate content directly after intense exercise (Clark et al. 2004). Therefore, it is presently unknown if high intensity training encompassing repeated 30-sec sprint intervals, in combination with reduction in volume, changes muscle anaerobic energy turnover during submaximal exercise in trained subjects.

Thus, the aims of the present study, using a training intervention consisting of repeated 30-sec sprinting and aerobic high intensity training for a group of trained cyclists, were to examine (1) whether intensified training increases the amount of oxidative enzymes in FT muscle fibers as well as speeds 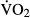 kinetics and reduces the 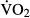 slow component during intense exercise; (2) if the anaerobic energy turnover during intense exercise was reduced by training. We hypothesized that the intensified training would increase the oxidative capacity of FT fibers resulting in faster 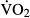 kinetics during intense exercise, but not during moderate intensity exercise, as well as reduce the 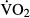 slow component and anaerobic energy turnover during intense exercise.

## Methods

### Subjects

Eight trained male cyclists with an average (±SD) age, weight, and 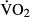-max of 33 ± 8 years, 81 ± 8 kg, and 4.8 ± 0.3 L min^−1^ or 59 ± 4 mL min^−1^ kg^−1^, respectively, were recruited for the study. Prior to participating in the study, the subjects had trained/competed ∼three to five times each week for at least 3 years. The study procedures were approved by the local ethical committee of the capital region of Copenhagen (Region Hovedstaden) and all subjects received written and oral information about the study procedures and gave their written informed consent to participate in the study in accordance with the Helsinki declaration.

### Experimental design

The subjects carried out a 7-week high-intensity training (HIT) intervention (see later) from October to December just after the season had finished, hence subjects were expected to be fit and in a physical stable condition. Both before and after HIT the subjects carried out two main experiments (EXP1 & EXP2) to evaluate changes in the metabolic response and 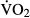 kinetics during submaximal exercise (<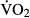-max). The subjects and training intervention were the same as in a study focusing on adaptations of ion transport proteins, ion kinetics, and performance during repeated high intensity exercise (Gunnarsson et al. 2013).

### Training

**Figure 1:**
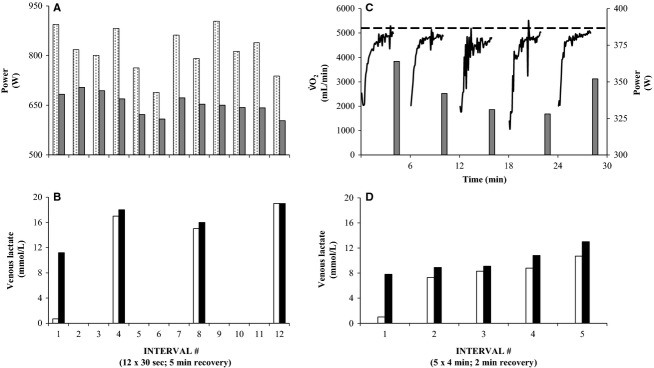
The physiological response during a training session with 12 × 30-sec sprint intervals separated by 5 min of recovery (A & B) and a session with 5 × 4 min intervals separated by 2 min of recovery (C & D) for one subject having a 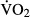-max of 5.2 L min^−1^ (hatched line). Peak power (dotted bars), mean power (gray bars), and pulmonary 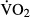 (full line) is shown for each interval (top) together with lactate (bottom) from an antecubital vein before (open bars) and after (full bars) intervals. Each of the two training sessions was performed indoor using the bike ergometer and 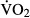-system described in the methods section.

Subjects performed four supervised training sessions per week on their own bikes on public roads. Training was performed as 12 × 30-sec uphill (∼6% gradient) maximal sprints interspersed with 4–5 min low intensity recovery (SPR; 2.5× week^−1^) resulting in a 1:8–10 work rest ratio, and 5 × ∼4 min aerobic high intensity intervals separated by ∼2 min of rest (AEH; 1.5× week^−1^) on a flat 2.5 km course with a work rest ratio of 2:1. In a training week day 1 was recovery, AEH was performed on day 2, SPR on day 3, recovery on day 4, SPR on day 5, recovery on day 6, and finally SPR or AEH on day 7 in alternate weeks. To ensure maximal effort during training drafting was not allowed and both SPR (1 vs. 1) and AEH (mass start) was performed in a competitive manner with the objective of finishing first. Heart rate (HR) was measured in 5-sec intervals during training (Polar Team Edition, Finland). Peak-HR during each SPR interval was 90 ± 4% of HR-max and average-HR during each AEH interval was 89 ± 2% of HR-max. The physiological response from one of the subjects during a SPR and an AEH training session is shown in Fig.1. Weekly volume was ∼240 min during HIT (15 min SET [∼6%], 30 min AEH [∼12%], 135 min low intensity recovery between intervals [56%] and ∼60 min moderate intensity [25%] as transport to and from training). This amounted to a ∼50% reduction of the training volume being 472 ± 153 min week^−1^ before HIT (0–60% HR-max [29%], 60–70% HR-max [24%], 70–80% HR-max [19%], 80–90% HR-max [21%], 90–95% HR-max [6%], 95–100% HR-max [1%]).

### Exercise testing

All testing was performed on a mechanically braked ergometer bike (Monark 839E, Varberg, Sweden) with the subjects using their own pedals and specific geometric setup which was maintained throughout the study. Subjects were instructed not to perform any training the day before a testing session, and maintain the same food intake and abstain from intake of caffeine on days of testing.

During the first visit to the laboratory during the competitive season subjects performed an incremental test starting out at 100 W with increments of 25 W min^−1^ until exhaustion. Pulmonary 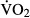 was measured breath by breath (Oxycon Pro, Viasys Healthcare, CareFusion, Rolle, Switzerland) and VO_2_-max and HR-max was determined as the highest value over a 30-sec period. In addition incremental test peak power output was calculated as:

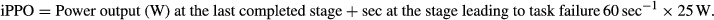
iPPO was 409 ± 24 W. Before and after HIT changes in pulmonary 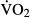 kinetics was investigated using repeated exercise transitions. Subjects performed both moderate (MOD: 50% iPPO, 205 ± 12 W) and intense (INT: 70% iPPO, 286 ± 17 W) exercise with 2 min at 20 W preceding all intervals. The absolute exercise intensity was maintained throughout the study. Both prior to and following HIT subjects performed four transitions with MOD each lasting 6 min (EXP1, EXP2, and two additional transitions on separate days) and three transitions with INT each lasting 6 min (EXP1, EXP2, and an additional transition on a separate testing day) as well as two transitions with INT lasting 3 min (EXP1, EXP2). Pulmonary 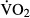 was measured breath by breath. To determine 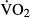 kinetics, errant breaths, defined as any value lying more than 4 SDs away from the local mean (e.g., due to swallowing and coughing) were initially removed. Then the 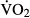 responses in each intensity domain were linearly interpolated to give 1-sec values, and then averaged. The initial cardiodynamic component was ignored by eliminating the first 20 sec of data after the onset of exercise.

MOD was modeled via a mono exponential function:

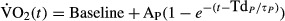


INT was modeled via a bi-exponential function:

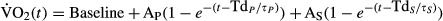
with 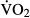 (*t*) being 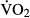 to a given time (sec). 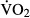 baseline was calculated as average 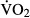 from 30 to 90 sec of the 120 sec baseline cycling at 20 W. A_P_, Td_P_, and *τ*_P_ are the amplitude, time delay, and time constant, respectively, for the primary (P) response. A_S_, Td_S_, and *τ*_S_ are the truncated amplitude, time delay, and time constant, respectively, for the slow component (S). An iterative process was used to determine the best fit of the curve. The relative VO_2_ slow component was determined as the ratio between A_S_ and average VO_2_ during the last 2 min of exercise.

Mechanical gross efficiency (GE) was calculated for both MOD and INT during the last 2 min of exercise using the formula

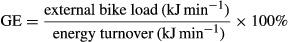


Energy turnover was estimated as 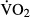 (L min^−1^) × energetic value of oxygen (kJ L^−1^) with the latter being calculated from the measured respiratory exchange ratio (RER) thus taking into account the different energy yield from oxidation of carbohydrate and fat. In the event of RER exceeding 1.0 a value of 1.0 was used in the calculation.

### Main experiments

Two experimental days (EXP1 & EXP2) were performed both prior to and after HIT to quantify muscle metabolites during INT. Subjects arrived to the laboratory in the morning after consuming a light breakfast.

On both EXP1 and EXP2 a biopsy at rest was collected from m. vastus lateralis under local anesthesia using a Bergstrom needle with suction. One part was frozen immediately in liquid nitrogen within ∼10 sec for analysis of metabolites, enzyme activity, and protein content. Another part of the biopsy was embedded in tissue tec (Sakura Finetek, Netherlands) for histochemical analysis. Initially, 6 min of MOD was performed followed by rest for 30 min. To evaluate changes in muscle metabolism in response to the HIT-period a muscle biopsy was obtained following 6 min of INT on EXP1 and following 3 min of INT on EXP2. Following 60 min of rest, 3 min of INT was performed on EXP1 and following 30 min of rest, 6 min of INT was performed on EXP2.

### Muscle analysis

All muscle samples were stored at −80°C until analyzed.

#### Muscle metabolites

The muscle biopsies taken at rest and after 3 and 6 min of INT were analyzed for levels of lactate and creatine phosphate (CP) using fluorometric methods (Lowry and Passonneau 1972).

#### Maximal enzyme activity

In part of the muscle biopsy obtained at rest maximal enzyme activity of citrate synthase (CS), 3-Hydroxyacyl CoA dehydrogenase (HAD), phosphofructokinase (PFK), and lactate dehydrogenase (LDH) was quantified in muscle homogenates after freeze drying and removal of fat and connective tissue using fluorometric methods (Fluoroscan Ascent, Thermo Scientific, Waltham, MA) (Lowry and Passonneau 1972).

#### Protein expression in muscle homogenate lysates

Approximately 3 mg freeze dried muscle tissue was split in two for double protein determination to increase measuring sensitivity and then homogenized and centrifuged to exclude non dissolved structures, as previously described (Bangsbo et al. 2009). Total protein concentrations were determined in each sample using BSA standards (Pierce, IL) and the lysates were then diluted in 6× Laemmli buffer and ddH_2_O to reach equal protein concentration before protein expression of CS, cytochrome c oxidase complex 4 (COX-4) and PFK were determined by western blotting. For subsequent analysis the average value was calculated from the two samples.

#### Protein expression in segments of human single muscle fibers

The determination of fiber type-specific changes in protein expression for CS, COX-4, and PFK were performed as previously described Thomassen et al. (2013) with minor changes. After freeze drying the muscle tissue samples (7–10 mg dry weight, *n* = 16) for 48 h, segments of single fibers were dissected under a microscope and stored in single microfuge tubes. The average size of the segments collected were roughly determined by measuring the lengths of the fiber under a microscope (1.4 ± 0.3 mm, mean ± SD, *n* = 398). Before SDS-PAGE 18 *μ*L 6× Laemmli buffer (0.7 mL 0.5 mol L^−1^ Tris-base, 3 mL glycerol, 0.93 g DTT, 1 g SDS and 1.2 mg bromophenol blue) diluted (1:3, v:v) in ddH_2_O was added to each fiber and incubated for 1 h at room temperature.

In order to have equal number of fibers in the different groups, all single fiber segments were first fiber typed before the analysis of the protein of interest. About 5 *μ*L of the samples were loaded on a 26 well Tris-Tricine 4–15% Criterion gels (Bio-Rad Laboratories, Solna, Sweden) and by western blotting characterized as either slow twitch (ST) or fast twitch (FT) muscle fibers by use of antibodies specific for myosin heavy chain (MHC) type I (ST fibers) and MHC type II (FT fibers) as well as the FT-specific SERCA1 protein (Thomassen et al. 2013). Antibodies used were ST fibers: 0.5 *μ*g·mL^−1^, mouse monoclonal IgM, A4.840, Developmental Studies Hybridoma Bank (DSHB), University of Iowa, USA; FT fibers: 2 *μ*g·mL^−1^, mouse monoclonal IgG, A4.74, DSHB, both developed by Dr Blau, and SERCA1: 0.1 *μ*g·mL^−1^, mouse monoclonal, MA3-912, Thermo Scientific. From this prefiber typing, 160 fibers were selected (1.5 ± 0.3 mm) to the final analyses, including 20 from each subject with five fibers from each of the four groups: ST pre, ST post, FT pre, and FT post. The remaining 13 *μ*L of the given selected fiber segments were then loaded onto additional gels.

#### Western blotting

For both segments of single muscle fibers and muscle homogenate lysates proteins were separated by SDS-PAGE (55 mA per gel and maximum 150 V) for ∼120 min and then semi-dry transferred to a PVDF membrane (Millipore A/S, Copenhagen, Denmark) for 120 min at 70 mA per gel and maximum 25 V. After protein transfer, gels including single fibers were incubated for 1 h in Coomassie stain, including 0.3% Coomassie Brilliant Blue R (Sigma-Aldrich, Copenhagen, Denmark), 40% ethanol (96%), 10% Acetic acid (Merck, Copenhagen, Denmark) and 49.7% ddH_2_O. Gels were then destained in ddH_2_O overnight and imaged using a ChemiDoc MP Imaging System (Bio-Rad Laboratories). These Coomassie-stained posttransferred gels were used to determine the amount of protein in each lane, based on the MHC (∼200 kDa) bands (Murphy et al. 2006). Membranes were blocked in Tris-buffered saline including 0.1% Tween-20 (TBST) with either 2% skimmed milk or 3% BSA for 1 h and then incubated with primary antibodies over night. After 2 washes in TBST, horseradish peroxidase-conjugated secondary antibody (DAKO, Glostrup, Denmark) diluted 1 to 5000 in TBST with addition of either 2% skimmed milk or 3% BSA was added, following which the membranes were washed in TBST (3 × 15 min). Bands were visualized using chemiluminescent detection (single fiber analysis using Super Signal West Femto Maximum Sensitivity Substrate, Thermo Scientific, – muscle lysates using ECL, Millipore) and images were collected on a ChemiDoc MP Imaging System. For further analyses, the membrane was kept in TBST and re-incubated in a new primary antibody overnight, giving the opportunity to determine the expression of several proteins with different molecular weights on the same segment of fibers (Thomassen et al. 2013).

The membranes for single fiber analyses were divided into four pieces by cutting over 250 kDa, right below the 150 kDa and 75 kDa, above the 25 kDa and below the 10 kDa markers (All Blue and Dual Color, Bio-Rad Laboratories). The first and upper part was used to confirm the predetermined fiber type (ST and FT 200 kDa), the second part used for PFK (85 kDa) and SERCA1 (100 kDa), the third for CS (48 kDa) and Actin (42 kDa), and the fourth and lower part used for COX-4 (14 kDa).

Antibody details: Other antibodies used for protein expression determination were: PFK: 0.2 *μ*g·mL^−1^, mouse monoclonal, Sc166722; Santa Cruz Biotechnology, Dallas, TX; CS: 0.33 *μ*g·mL^−1^, rabbit polyclonal, ab96600; Abcam, Cambridge, UK; COX-4: 0.2 *μ*g·mL^−1^, mouse monoclonal, Sc58648; Santa Cruz Biotechnology.

#### Data treatment

In total, 160 segments of human skeletal muscle single fibers from vastus lateralis were used for the final protein expression determination in ST and FT fibers. On each gel 5 ST and 5 FT fibers from a resting pre-HIT muscle and 5 ST and 5 FT fibers from a resting post-HIT muscle from the same individual were loaded. Given the small size of segments of individual fibers, it was not possible also to determine the total protein concentration in each sample prior to sample loading. Consequently, different amounts of protein were loaded in each well. In order to compare the specific protein expression between fiber samples, MHC on the post-transferred gel was quantified and it was deemed that an equal proportion of total protein was transferred to the membrane independent of total amount loaded. Thus, the signal on the posttransferred Coomassie stained gel was used for normalization of the densities of the protein of interest, as previously demonstrated as a reliable measure (Murphy et al. 2006; Thomassen et al. 2013).

The signal intensity for each protein of interest was first normalized to the mean intensity of all single human fiber bands for that protein on the gel. Afterward data were normalized to the total amount of protein in each sample determined by Coomassie staining of the remaining MHC. In order to compare fibers loaded on different gels single values were normalized to the mean of ST pre-HIT in the single fiber analysis and pre-HIT in the muscle homogenate analysis. Finally, in order to have a normal distribution of the data, the ratios were log transformed before the statistical analysis. For clarity the graphical presentation of the results are displayed as ratios relative to ST pre-HIT for single fiber data and from the backtransformed log values relative to pre-HIT for muscle homogenate.

#### Capillary density

Capillarization was analyzed using fluorescence microscopy. Transverse sections of the muscle biopsies were cut at a thickness of 8 *μ*m and placed onto glass slides and first treated with Biotinylated Ulex Europaeus Agglutinin I (Vector Laboratories, Burlingame, CA) and later with Streptavidin 1:200 (Dako, Glostrup, Denmark). Subsequently pictures were taken of the muscle samples and analyzed on a computer (ImageJ) for capillary to fiber ratio.

### Statistics

Before and after HIT changes in 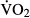 kinetics, capillary to fiber ratio, muscle metabolites at rest and after 3 and 6 min of exercise as well as enzyme activity and protein level in homogenates were examined with a Student’s paired *t*-test. Also a paired *t*-test was used to evaluate if *τ* of the primary phase differed between MOD and INT both pre- and post-HIT. Changes in segments of single muscle fibers were evaluated using a two-way ANOVA General Linear Model (ST vs. FT fibers and pre vs. post-HIT as factors). The five ST fibers were averaged to yield one value for each subject both pre- and post-HIT and the same approach was made for the five FT fibers. If significant main effects or an interaction were observed, then a student Newman-Keuls post hoc analysis was performed to identify the specific differences in protein expression within fiber types. Correlations between 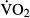 kinetics (*τ* of the primary response during MOD and INT and the relative VO_2_ slow component) and maximal aerobic enzyme activity and the capillary to fiber ratio was evaluated using a one-tailed test with the a-priory hypothesis that fast 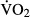 kinetics and a minor 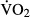 slow component would be associated with a high aerobic enzyme activity and capillary to fiber ratio.

## Results

### Muscle adaptations

**Figure 2:**
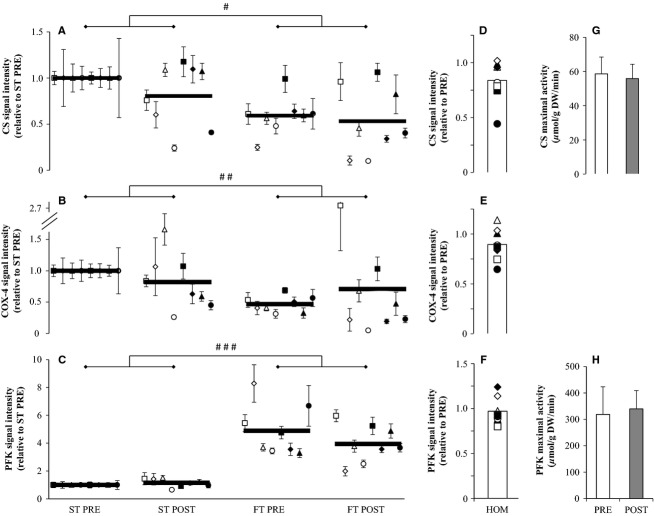
Enzyme content following 7 weeks of high intensity and reduced volume training in trained cyclists (*n* = 8). The average content (thick lines) of CS (A), COX-4 (B), and PFK (C) before (PRE) and after (POST) the intervention are shown in slow-twitch (ST) and fast-twitch (FT) fibers together with individual values (average of five fibers ± SEM at each time point) which have been normalized to ST PRE for all enzymes. Average and individual protein content measured in homogenates (HOM) are also shown for CS (D), COX-4 (E), and PFK (F) as well as maximal activity for CS (G) and PFK (H). For clarity the graphs A-F display ratio data but the statistical analysis was based on log data. ^#^*P* < 0.05, ^##^*P* < 0.01, ^###^*P* < 0.001; significant difference between ST and FT fibers.

**Figure 3:**
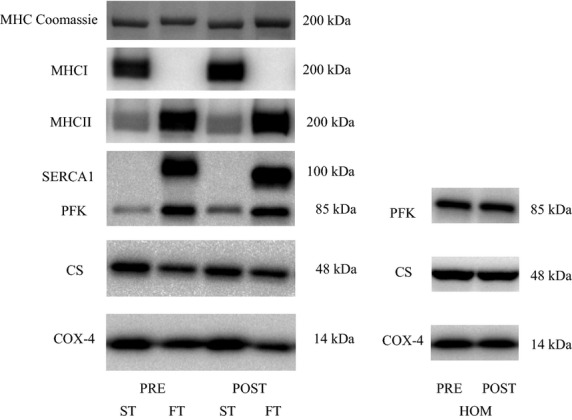
Representative western blots of the proteins investigated in slow-twitch (ST), fast-twitch (FT), and muscle homogenate (HOM) before (PRE) and after (POST) 7 weeks of high intensity and reduced volume training in trained cyclists. See methods section for details.

After HIT no overall effect of the intervention was observed for the amount of CS (*P* = 0.12; Fig.2A), COX-4 (*P* = 0.20; Fig.2B) and PFK (*P* = 0.70; Fig.2C) in segments of ST and FT fibers. An overall effect was found for fiber type for CS (*P* = 0.01), COX-4 (*P* = 0.007) and PFK (*P* < 0.001) since protein content in ST fibers was higher than FT fibers for CS (*P* = 0.016 pre-HIT and *P* = 0.007 post-HIT) and COX-4 (*P* = 0.012 pre-HIT and *P* = 0.01 post-HIT) and lower for PFK (*P* < 0.001 pre and post-HIT). Before HIT the content in ST fibers of CS and COX-4 was on average 53% and 41% higher than in FT fibers whereas PFK was ∼389% higher in FT fibers with respective values after HIT being 34%, 15% and ∼241%. In muscle homogenates both CS (*P* = 0.07; Fig.2A) and COX-4 (*P* = 0.10; Fig.2B) tended to be lower after compared to before HIT with an average decrease in protein content of 16% and 11%, respectively. PFK remained unchanged (*P* = 0.45; Fig.2C) with an average decrease of 3%. Representative Western blots of the proteins investigated are displayed in Fig.3.

After HIT the maximal enzyme activity was not changed relative to before the intervention for CS (56 ± 8 vs. 59 ± 10 *μ*mol g DW^−1^ min^−1^; *P* = 0.10), HAD (27 ± 6 vs. 29 ± 3 *μ*mol g DW^−1^ min^−1^; *P* = 0.41), LDH (131 ± 27 vs. 113 ± 27 *μ*mol g DW^−1^ min^−1^; *P* = 0.14) and PFK (340 ± 69 vs. 318 ± 105 *μ*mol g DW^−1^ min^−1^; *P* = 0.49).

**Figure 4:**
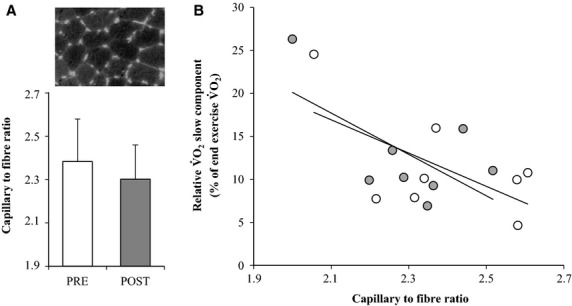
Muscle capillary to fiber ratio (A) before (PRE; open bars) and after (POST; closed bars) 7 weeks of high intensity and reduced volume training in trained cyclists (*n* = 8) with insert picture showing staining of capillaries in a representative subject. Values are means ± SD. Association between the capillary to fiber ratio and the relative 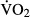 slow component (B) before (open symbols, *r*^2^ = 0.38; *P* > 0.05) and after (closed symbols, *r*^2^ = 0.39; *P* < 0.05) 7 weeks of high intensity and reduced volume training in trained cyclists (*n* = 8).

Following HIT no changes relative to before the intervention were observed in the capillary to fiber ratio (2.30 ± 0.16 vs. 2.38 ± 0.20; *P* = 0.13) (Fig.4A).

### 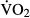 kinetics

**Figure 5:**
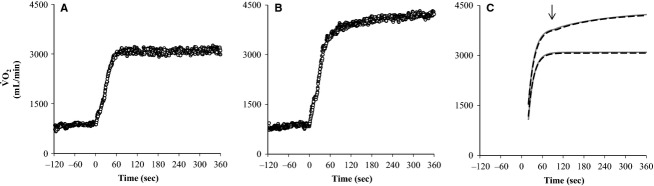
Pulmonary oxygen uptake (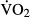) following 7 weeks of high intensity and reduced volume training in trained cyclists (*n* = 8) during moderate (MOD; A) and intense (INT; B) cycling with the modeled responses shown (C) before (PRE; open symbols and hatched lines) and after (POST; filled symbols and solid lines) the intervention. The arrow in panel C indicates the onset of the 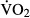 slow component during INT (103 and 95 sec on average PRE and POST HIT) being superimposed on the primary 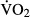 response.

No significant differences following HIT were observed in the 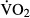 response during MOD (Fig.5) and in all modeling parameters (Table1) as *τ* was unchanged (*P* = 0.67) together with absolute 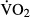 (*P* = 0.39), RER (*P* = 0.63) and GE (*P* = 0.39) averaged over the last 2 min of exercise.

**Table 1:** Pulmonary 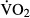 kinetics modeling parameters during moderate (MOD) and intense (INT) cycling before (PRE) and after (POST) 7 weeks of high intensity and reduced volume training in trained cyclists (*n* = 8). Changes in pre and post were evaluated with a paired *t*-test

	PRE	POST
MOD		
Baseline (mL min^−1^)	865 ± 96	849 ± 60
Td_P_ (sec)	17.5 ± 3.1	18.3 ± 2.9
*τ*_P_ (sec)	15.9 ± 2.4	15.4 ± 2.5
A_P_ (mL min^−1^)	2209 ± 119	2249 ± 144
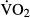 4–6 min (mL min^−1^)	3083 ± 172	3110 ± 165
RER 4–6 min	0.95 ± 0.02	0.95 ± 0.01
GE 4–6 min (%)	19.1 ± 0.7	18.9 ± 0.7
Cadence (rounds min^−1^)	92 ± 9	92 ± 8
INT		
Baseline (mL min^−1^)	870 ± 65	824 ± 53
Td_P_ (sec)	15.2 ± 1.5	15.3 ± 2.3
*τ*_P_ (sec)	18.2 ± 2.5	17.2 ± 2.9
A_P_ (mL min^−1^)	2931 ± 240	2990 ± 186
Td_S_ (sec)	103 ± 30	95 ± 56
*τ*_S_ (sec)	141 ± 60	181 ± 111
A_S_ (mL min^−1^)	479 ± 264	539 ± 257
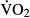 4–6 min (mL min^−1^)	4153 ± 234	4180 ± 184
RER 4–6 min	1.03 ± 0.04	1.01 ± 0.02
GE 4–6 min (%)	19.6 ± 0.9	19.5 ± 0.7
Cadence (rounds min^−1^)	93 ± 6	94 ± 6

Values are means ± SD. Baseline (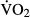 before onset of exercise). Time delay (Td), time constant (*τ*), amplitude (A) for the primary response (_P_), and the slow component (_S_). Absolute oxygen uptake (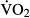), respiratory exchange ratio (RER), and gross efficiency (GE) in the last 2 min of exercise from 4–6 min.

The 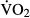 response during INT was also unaffected by HIT (Fig.5) as were all modeling parameters (Table1) including *τ* of the primary response (*P* = 0.32), the absolute (*P* = 0.26) and relative (*P* = 0.25) size of the 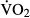 slow component of the secondary response together with absolute 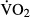 (*P* = 0.52), RER (*P* = 0.13) and GE (*P* = 0.46) averaged over the last 2 min of exercise.

*τ* during MOD and INT was not different neither pre (*P* = 0.12) nor post-HIT (*P* = 0.22).

### Correlations

Maximal activity of CS (*r*^2^ = 0.002–0.18; *P* > 0.05) and HAD (*r*^2^ = 0.0001–0.16; *P* > 0.05) did not correlate with *τ* of the primary response during MOD and INT both before and after HIT. Neither did CS correlate with the relative size of the 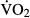 slow component (*r*^2^ = 0.38 and 0.30 before and after HIT; *P* > 0.05) as was the case for HAD (*r*^2^ = 0.12 and 0.30 before and after HIT; *P* > 0.05). The capillary to fiber ratio was associated with *τ* during MOD before (*r*^2^ = 0.90; *P* < 0.001) but not after HIT (*r*^2^ = 0.03; *P* > 0.05) and no association was present during INT (*r*^2^ = 0.10–0.14; *P* > 0.05). An association between the capillary to fiber ratio and the relative size of the 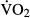 slow component (Fig.4B) was present following HIT (*r*^2^ = 0.39; *P* < 0.05) but not before HIT (*r*^2^ = 0.38; *P* > 0.05).

### Muscle metabolites

**Figure 6:**
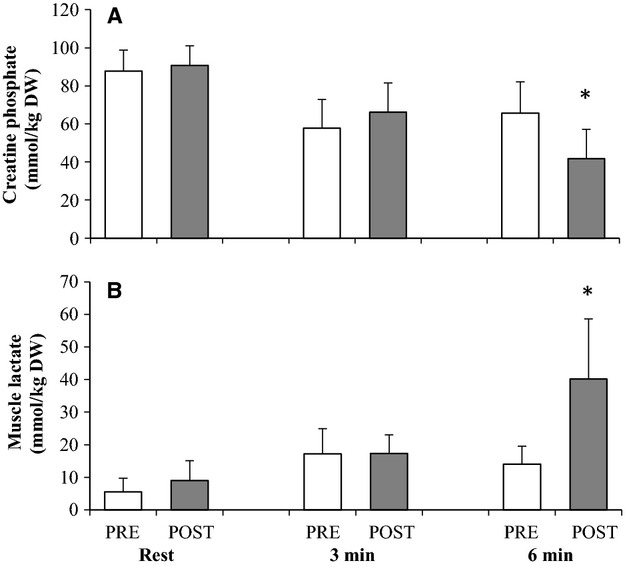
Muscle creatine phosphate (A) and lactate (B) at rest and after 3 and 6 min of intense cycling before (PRE; open bars) and after (POST; closed bars) 7 weeks of high intensity and reduced volume training in trained cyclists (*n* = 7). Values are means±SD. *P < 0.05; significant difference between PRE- and POST-values.

Muscle CP (*n* = 7) was not changed by HIT at rest (∼90 mmol g DW^−1^ min^−1^; *P* = 0.60) and after 3 min of INT (∼60 mmol g DW^−1^ min^−1^; *P* = 0.41), but after HIT it was lower at 6 min of exercise than before HIT (42 ± 15 vs. 66 ± 17 mmol kg DW^−1^; *P* = 0.011) (Fig.6A).

Muscle lactate (*n* = 7) was not changed by HIT at rest (∼7 mmol g DW^−1^ min^−1^; *P* = 0.18) and after 3 min of INT (∼17 mmol g DW^−1^ min^−1^; *P* = 0.95), but at 6 min muscle lactate was higher after compared to before HIT (40.2 ± 18.4 vs. 14.1 ± 5.5 mmol kg DW^−1^; *P* < 0.012) (Fig.6B).

## Discussion

The major findings in the present study were that a period of reduced and intensified training performed by trained cyclists did not elevate the protein content of oxidative enzymes in FT fibers. Furthermore, no components of pulmonary 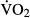 kinetics were changed, whereas higher muscle lactate accumulation and lower level of CP were observed during intense cycling after compared to before the intervention period.

Contrary to our hypothesis the content of CS and COX-4 in the FT fibers was not elevated after the intervention period in the form of volume reduced and intensified training. The high exercise intensity used in HIT, in particular the repeated sprint training, was chosen in order to activate all FT fibers with this type of exercise being a potent stimulator of the signal cascades leading to adaptations of the muscular oxidative system (Psilander et al. 2010; Little et al. 2011). Thus, it was unexpected that no change in oxidative enzyme content was present in FT fibers after the training period. One explanation may be that the cyclists during their normal training and competition prior to the intervention period, routinely engaged most of their muscle fibers, and that “extra” FT fibers activated during the training in the intervention period were only fibers from the highest order motor units. Then, the FT fibers in “lower” order motor units were recruited less due to the reduction in training volume from ∼470 to ∼240 min week^−1^. In support of this, it has been shown that, even at 65% (Scribbans et al. 2014) and 80% (Krustrup et al. 2004b) of 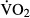-max, a significant number of FT fibers are recruited. In the present study a considerable part of the training prior to HIT was performed near 80% 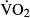-max (∼28% of total training time or ∼130 min week^−1^ was carried out with a heart rate above 80% of HR-max). Furthermore, detraining in trained athletes is known to reduce content and maximal activity of oxidative enzymes (Chi et al. 1983; Christensen et al. 2011). Thus, it may be that the protein content of oxidative enzymes was reduced in the motor units controlling FT fibers lower in the fiber hierarchy due to the reduction in training. This may also explain the overall tendency for a drop in protein content and maximal activity of CS and COX-4 measured in muscle homogenates.

The single fiber data of CS and COX-4 showed a large variation in oxidative enzyme protein content within each fiber type as evidenced by the high standard error for many of the cyclists (Fig.2A and B). This is in agreement with the pioneering work by Lowry et al. (1978) showing a large range in maximal enzyme activity in single fibers, but in that study a separation between ST and FT fibers was not made. The observed difference in the content of muscle oxidative enzymes between individual fibers supports the proposed concept that motor units have a range from very fast to very slow 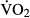 kinetics as well as different steady-state values (Koppo et al. 2004). Studies using trained subjects (Jansson and Kaijser 1977; Chi et al. 1983) and a longitudinal study on untrained subjects (Henriksson and Reitman 1976) have shown that the maximal activity of oxidative enzymes in FT fibers can be as high as in ST fibers. This was not the case in the present study, which may be due to a higher aerobic training status (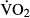-max: ∼70 mL min^−1^ kg^−1^) of the subjects in the studies showing similar levels in FT and ST fibers (Jansson and Kaijser 1977; Chi et al. 1983). Nevertheless, the combination of repeated 30-sec sprinting (2.5 × week^−1^) and aerobic high intensity training (1.5 × week^−1^) in the present study with a reduced training volume did not increase the oxidative enzyme content in FT fibers in already trained athletes with a 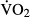-max of ∼60 mL min^−1^ kg^−1^. On the other hand, it has been shown that adding two weekly aerobic high intensity sessions (6 × ∼3 min with ∼1.5 min recovery), at the speed eliciting task failure in an incremental test, increased the content of LDH in the FT fibers after 6 weeks of training with a maintained training volume in well-trained runners (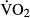-max: 67 mL min^−1^ kg^−1^) (Kohn et al. 2011). Therefore, it seems plausible that the training reduction in the present study can explain the lack of increase in oxidative protein content in FT fibers. Alternatively, training with repeated sprints may be less potent than aerobic high intensity training (3–5 min intervals at ∼90–100% 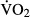-max) to increase protein content of oxidative enzymes in FT fibers in trained individuals, since the former type of training dominated in the present study.

Based on changes in blood and muscle lactate in trained individuals it does appear that different types of high intensity training can yield different outcomes. Accordingly, aerobic high intensity training (<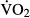-max; e.g., 3–5 min intervals) and a maintained training volume appears to lower muscle (Clark et al. 2004) and blood lactate (Acevedo and Goldfarb 1989; Kohn et al. 2011) during intense exercise (<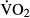-max), whereas training interventions with repeated 30-sec sprints in combination with a volume reduction does not lower blood lactate (Bangsbo et al. 2009; Iaia et al. 2009) and in the present study muscle lactate was higher after training. Future studies are needed to evaluate if the outcome on oxidative adaptations in FT fibers differs between different types of high intensity training which appears to be the case with regards to lactate levels. The role of the total training volume is considered of interest, thus either adding SET on top of the normal training or having SET substitute less intense training seems relevant.

Pulmonary 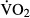 kinetics was not changed with HIT (Fig.5 and Table1). This finding differs from the faster 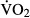 kinetics observed after a period of intense training in untrained subjects at both the muscular (Krustrup et al. 2004a) and pulmonary level (Bailey et al. 2009) with the latter study also reporting a reduced 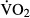 slow component. Despite the tendencies for a reduction in the content of CS and COX-4 as well as the maximal activity of CS in the present study, 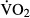 kinetics remained unchanged. These findings suggest that the level of oxidative enzymes does not limit the muscle oxygen utilization in the initial part of exercise, nor the development of the 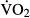 slow component. It should, however, be considered that the content and maximal activity of the oxidative enzymes was not significantly reduced, and it may be that endurance athletes have an excessive oxidative enzyme capacity allowing a modest drop without having an effect on 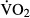 kinetics. The CS activity measured in muscle homogenate was ∼60 *μ*mol g DW^−1^ min^−1^, which is about twice as high as in untrained subjects (Krustrup et al. 2004a; Burgomaster et al. 2005, 2008) and somewhat higher than previous observations in trained endurance athletes with a similar 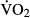-max as in the present study (Yeo et al. 2008; Bangsbo et al. 2009). In a study of trained soccer players 2 weeks without training resulted in a decrease in activity and content of oxidative enzymes, which was associated with slower 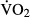 kinetics (Christensen et al. 2011). Thus, it cannot be excluded that the level of oxidative enzymes under some circumstances may become limiting for 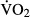 kinetics in trained individuals. Unlike previous findings in trained individuals (Koppo et al. 2004) the speed of the primary 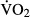 response was not significantly different between MOD (*τ*∼15.7 sec) and INT (*τ*∼17.7 sec) although five of eight subjects had larger *τ* in INT than in MOD both pre- and post-HIT. Thus, despite an expected larger recruitment of FT fibers in INT relative to MOD (Krustrup et al. 2004b) and the fact that FT fibers had markedly lower content of oxidative enzymes than ST fibers in the present study (Fig.2A and B), the 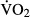 kinetics of the primary response in INT was not slower than MOD. This in turn suggests that at least in ST fibers there is an excess capacity of oxidative enzymes that does not impact on the speed of the VO_2_ response. Maximal oxidative enzyme activity of CS and HAD were poor predictors of fast 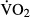 kinetics and the relative size of the 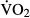 slow component showing that in trained individuals these enzymes appear to be of minor importance and other muscular variables needs to investigated. Of interest was the association between the capillary to fiber ratio and the relative size of the 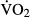 slow component after the HIT intervention and the marked tendency for an association before HIT (Fig.4). However, the association was mainly due to one subject having a low capillary to fiber ratio. Nevertheless, during intense exercise eliciting a slow component – likely due to recruitment of FT fibers (Jones et al. 2011) – a high capillary to fiber ratio could be speculated to lower blood mean transit time optimizing conditions for diffusion of oxygen which is supported by the finding of a reduced 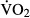 slow component during exercise inhaling an hyperoxic gas (Wilkerson et al. 2006).

The decrease in muscle CP and the increase in muscle lactate from 3 to 6 min in INT (∼85% 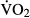-max) were larger after the HIT period (Fig.6). These findings suggest a larger anaerobic energy turnover in the last phase of INT. The activity of PFK was not elevated with the intervention period, neither when expressed as maximal activity or content, so it cannot explain the apparently greater rate of glycolysis (Spriet et al. 2000). The larger drop in CP indicates a higher accumulation of muscle ADP, which may have elevated the rate of glycolysis leading to a greater lactate production, but a larger ADP concentration would also be expected to stimulate the respiration but that was not the case in light of the unchanged 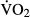 response. Alternatively, the tendency for a lower content and maximal activity of muscle oxidative enzymes (CS and COX-4) after the HIT period may have reduced the mitochondrial utilization of the produced pyruvate (Brooks 2000), thereby enhancing the lactate production catalyzed by LDH (Spriet et al. 2000). It is, however, unclear, why such changes did not occur in the first phase (0–3 min) of INT (Fig.6B). Nevertheless, it appears that the anaerobic energy flux during the last phase (3–6 min) of INT was higher after the intervention period, and thus, total energy turnover, as pulmonary VO_2_ was unaltered. Alternatively, this finding may reflect a greater imbalance between muscle CP and creatine and a change in the ratio between muscle lactate and pyruvate during exercise due to altered regulation. In moderately trained subjects (VO_2_-max: 49 mL min^−1^ kg^−1^) repeated sprint training, as used in the present study, resulted in lower muscle lactate and ATP concentrations after exercise at an intensity corresponding to 90% of 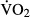-max. However, there were major differences between the present study and the one by Burgomaster and co-workers including a lower training status, and an increase in training volume and a higher maximal aerobic enzyme activity after the training period (Burgomaster et al. 2006) which may in part explain the different change in muscle lactate accumulation during intense exercise. The findings in the present study are in contrast to observations in a study also using well-trained cyclists (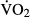-max: ∼65 mL O_2_ min^−1^ kg^−1^) who for a 3-week period added aerobic high intensity training (8 × 5 min ∼85% 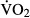-max) three times weekly to their normal training volume. Following the training period muscle lactate accumulation during intense exercise (∼85% 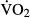-max) was reduced and during more moderate exercise (65% 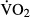-max) fat and carbohydrate oxidation was larger and lower, respectively (Clark et al. 2004). Content and maximal activity of oxidative enzymes were not reported, but was unchanged in trained cyclists in another study using the same type of training (Yeo et al. 2008). Taken together these findings suggest that the reduced amount of training in the present study is the major cause of the elevated anaerobic energy production during the intense submaximal work. Furthermore, the present study shows that anaerobic metabolism can be altered without a change in 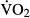 kinetics. Such dissociation between anaerobic and aerobic metabolism has also been reported in the exercise transient in studies using hyperoxia where CP utilization has been observed to be reduced (Vanhatalo et al. 2010) and the primary 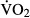 response appears to be unaffected (Wilkerson et al. 2006). Likewise, during steady-state conditions with moderate exercise higher CP and lower muscle lactate have been observed in hyperoxia (Stellingwerff et al. 2006) despite the 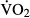 response being similar in both the exercise transient and in the stable phase of exercise (Wilkerson et al. 2006). Further studies are needed to evaluate how different training regimes influence anaerobic energy turnover during submaximal exercise.

The functional significance of the training period in the present study has been reported previously with regard to high intensity exercise performance in the form of improved performance in a repeated sprint test (6 × 20 sec) and in an exhaustive test lasting ∼4 min with the latter test being preceded by a 2-min preload with high intensity in which muscle lactate at the end of the preload also was elevated after HIT (Gunnarsson et al. 2013). This indicates that the apparently larger anaerobic muscle perturbation with HIT does not lower performance during intense exercise.

Each subject had five ST and FT fibers analyzed both pre and post-HIT (total of 20 fibers) and the average value of the five fibers for each time point (pre- and post-HIT) was used for further analysis. Such a low number may seem limiting, but a statistical difference was present between ST and FT fibers for all enzymes investigated in line with previous reports using pooled groups of ST and FT fibers to determine maximal enzyme activity (Essen et al. 1975; Essen-Gustavsson and Henriksson 1984; Schantz and Henriksson 1987) or content (Thomassen et al. 2013). Furthermore, the tendencies for a drop in homogenate protein content and maximal activity for CS and COX-4 and unchanged PFK levels were mirrored by the single fiber data. In addition the same three subjects who had a higher CS content in FT fibers after HIT also had elevated content of COX-4 (Symbols □, ■ and ▲on Fig.2). Taken together this suggests that the method is sensitive enough to detect possible changes in response to a training intervention.

In summary, the present study showed that the amount of CS and COX-4 in FT fibers and muscle homogenate as well as maximal activity of CS was not changed after a 7-week period of intensified training with a reduced volume in already trained cyclists. Furthermore, no change in pulmonary 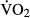 kinetics was observed during moderate (∼65% 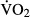-max) and intense (∼85% 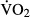-max) submaximal exercise. During intense cycling muscle CP levels were reduced and muscle lactate accumulation was elevated to a greater extent in the later part of exercise (3–6 min) following the training intervention without an altered 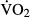. This could be interpreted to reflect a larger total energy turnover following the training intervention due to a greater anaerobic energy contribution to exercise or an altered regulation of anaerobic muscle metabolism.
